# Fluorescent protein tagging promotes phase separation and alters the aggregation pathway of huntingtin exon-1

**DOI:** 10.1016/j.jbc.2023.105585

**Published:** 2023-12-21

**Authors:** Nitin K. Pandey, Jobin Varkey, Anakha Ajayan, Gincy George, Jeannie Chen, Ralf Langen

**Affiliations:** 1 Physiology and Neuroscience, Zilkha Neurogenetic Institute, Keck School of Medicine, University of Southern California, Los Angeles, California, USA; 2 Biochemistry and Molecular Medicine, Zilkha Neurogenetic Institute, Keck School of Medicine, University of Southern California, Los Angeles, California, USA

**Keywords:** Huntington’s disease, polyglutamine, amyloid, liquid–liquid phase separation, protein aggregation, huntingtin exon-1, fibril

## Abstract

Fluorescent protein tags are convenient tools for tracking the aggregation states of amyloidogenic or phase separating proteins, but the effect of the tags is often not well understood. Here, we investigated the impact of a C-terminal red fluorescent protein (RFP) tag on the phase separation of huntingtin exon-1 (Httex1), an N-terminal portion of the huntingtin protein that aggregates in Huntington’s disease. We found that the RFP-tagged Httex1 rapidly formed micron-sized, phase separated states in the presence of a crowding agent. The formed structures had a rounded appearance and were highly dynamic according to electron paramagnetic resonance and fluorescence recovery after photobleaching, suggesting that the phase separated state was largely liquid in nature. Remarkably, the untagged protein did not undergo any detectable liquid condensate formation under the same conditions. In addition to strongly promoting liquid–liquid phase separation, the RFP tag also facilitated fibril formation, as the tag-dependent liquid condensates rapidly underwent a liquid-to-solid transition. The rate of fibril formation under these conditions was significantly faster than that of the untagged protein. When expressed in cells, the RFP-tagged Httex1 formed larger aggregates with different antibody staining patterns compared to untagged Httex1. Collectively, these data reveal that the addition of a fluorescent protein tag significantly impacts liquid and solid phase separations of Httex1 *in vitro* and leads to altered aggregation in cells. Considering that the tagged Httex1 is commonly used to study the mechanisms of Httex1 misfolding and toxicity, our findings highlight the importance to validate the conclusions with untagged protein.

Membraneless organelles (*e.g*., P granule, stress granule, and nucleoli) have gained significant attention due to their involvement in numerous cellular processes (1, 2, 3, 4). They are formed by biomolecules (including proteins and nucleic acids) through liquid–liquid phase separation (LLPS). The contents of these organelles are in dynamic equilibrium with the surroundings, and their formation is regulated by factors such as protein concentration, ionic strength, pH, cofactors, and posttranslational modifications (5, 6, 7, 8, 9). Several proteins implicated in neurodegenerative diseases, such as FUS, TDP-43, Tau, α-synuclein, and huntingtin, can undergo LLPS. In many of these proteins, LLPS can act as an intermediate during amyloid fibril formation, which is a common pathological hallmark of many neurodegenerative diseases (10, 11, 12, 13, 14, 15, 16).

A convenient way to monitor protein LLPS in cellular models is to employ fluorescent protein tags. Apart from LLPS, fluorescent protein tags have also been used extensively to study toxicity and disease mechanisms of many amyloid proteins, including huntingtin. Thus, knowing whether fluorescent protein tags can affect phase separation and aggregation behavior of these proteins is important but remains understudied. Here, we address this question for the huntingtin protein, which is involved in Huntington’s disease (HD). HD is caused by aberrant polyQ expansion (>35Q) in the first exon of the huntingtin protein (Httex1) (17, 18, 19), which is directly correlated with increased aggregation propensity and fibril formation (20). Httex1 contains three individual domains, N17, polyQ, and proline-rich domain (PRD). The N17 and expanded polyQ regions drive aggregation (21, 22, 23, 24), while the PRD is generally considered to be inhibitory (25, 26, 27, 28). Many studies have used Httex1 variants with fluorescent protein tags like GFP or RFP to explore misfolding mechanisms, formation of inclusions, and the toxicity of different conformers present in the misfolding pathways (29, 30, 31, 32, 33, 34, 35, 36, 37, 38, 39, 40, 41). Importantly, all studies showing that Httex1 can undergo LLPS have been performed using fluorescent protein tags (36). However, as with many proteins, the influence of the bulky protein tags on aggregation and phase separation has not been studied.

To address this question, we investigated the effect of a C-terminal RFP moiety on LLPS and aggregation of Httex1. We found that Httex1 had only a modest ability to undergo LLPS, requiring both the presence of a crowding agent and an RFP tag. No LLPS could be detected in the untagged protein. Rather, untagged Httex1 slowly transitioned into fibrils. The LLPS induced by the RFP tag furthermore strongly enhanced fibril formation and significantly altered aggregation in cells. Our results underscore the influence that protein tags can have on phase separation and aggregation of huntingtin, warranting similar investigations of other phase-separating proteins.

## Results

### RFP-tagged Httex1(Q25) undergoes phase separation in the presence of crowding agent

**Figure 1:**
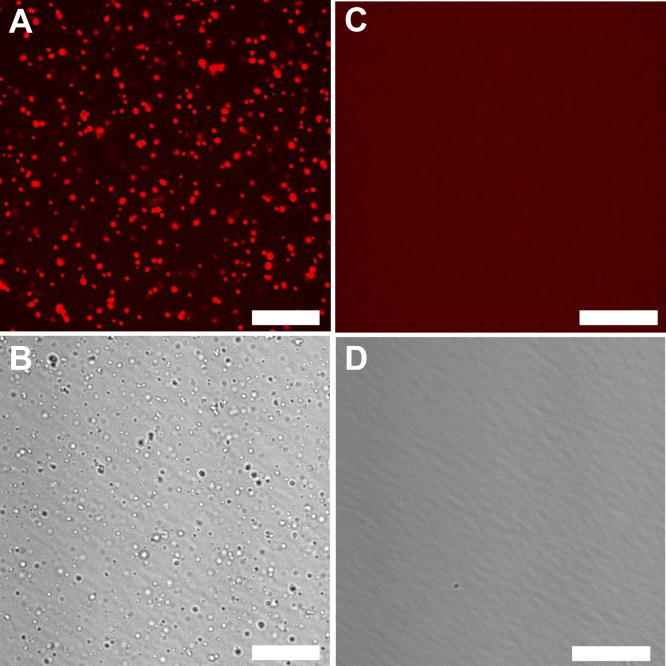
**Phase separation of Httex1(Q25)-RFP is promoted by crowding agents.***A*–*D*, freshly prepared Httex1(Q25)-RFP was imaged in the presence (*A* and *B*) or in the absence (*C* and *D*) of 10% PEG. Fluorescence imaging was used in *A* and *C*, whereas differential interference contrast was used in *B* and *D*. Phase separation can be observed in the presence of 10% PEG at 100 μM protein (*A* and *B*). Little or no evidence of phase separation was observed in the absence of PEG (*C* and *D*). The scale bars are 20 μm. Images are representative of at least three independent experiments. Httex1, huntingtin exon-1; Httex1(Q25)-RFP, RFP-tagged Httex1 with 25Qs; PEG, polyethylene glycol; RFP, red fluorescent protein.

To evaluate the factors that promote Httex1 LLPS, we first compared the phase separation of C-terminally RFP-tagged Httex1 with 25Qs (Httex1(Q25)-RFP) in the presence or absence of polyethylene glycol (PEG), a crowding agent commonly used to promote phase separation (5, 42, 43). Fluorescence microscopy revealed the formation of large, micron-sized phase-separated structures immediately following the reconstitution in the presence of PEG (Fig. 1*A*). The rounded shape of these phase-separated species was suggestive of LLPS, and they could also be detected by differential interference contrast (DIC) imaging, without the need for fluorescence (Fig. 1*B*). In contrast, no phase separation was observed by fluorescence imaging (Fig. 1*C*) or DIC (Fig. 1*D*) without PEG, as Httex1(Q25)-RFP largely gave rise to a diffuse red background. These results are consistent with previous reports showing that crowding agents strongly promote LLPS of GFP-tagged Httex1, which only has a low propensity to phase separate in the absence of crowding agents (36).

### Phase separation causes modest reduction in Httex1(Q25)-RFP dynamics

**Figure 2:**
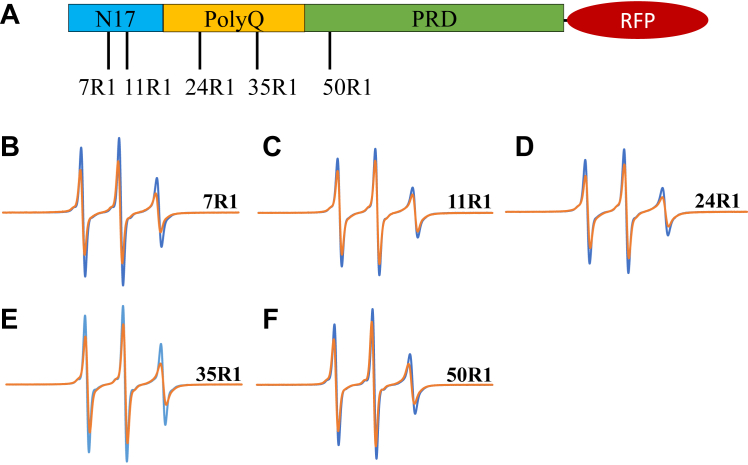
**Site-specific mobility changes associated with Httex1(Q25)-RFP phase separation.***A*, schematic representation of the Httex1 domain structure showing the locations of the spin-labeled sites used in the EPR experiments. *B*–*F*, continuous wave X-band EPR spectra of Httex1(Q25)-RFP spin-labeled at the following positions: (*B*) 7R1, (*C*) 11R1, (*D*) 24R1, (*E*) 35R1, and (*F*) 50R1. Spectra were recorded in the absence (*blue*) or presence (*orange*) of 10% PEG. The scan width was 100 G. All spectra were normalized to the same number of spins. Spectra are representative of at least three independent measurements. Httex1, huntingtin exon-1; Httex1(Q25)-RFP, RFP-tagged Httex1 with 25Qs; PEG, polyethylene glycol; RFP, red fluorescent protein.

To monitor the structure and dynamics upon phase separation, we generated spin-labeled derivatives of Httex1(Q25)-RFP and recorded their continuous wave electron paramagnetic resonance (EPR) spectra in the absence or presence of PEG. Spin labels were introduced in the N17 (7R1 and 11R1), the polyQ (24R1 and 35R1), and the PRD (50R1) region (Fig. 2*A*). The addition of PEG reduced the spectral amplitude for all derivatives, which was indicative of reduced mobility under conditions of phase separation (Fig. 2, *B*–*F*). It is commonly observed that phase separated states are in equilibrium with the monomeric proteins from which they arise. Consistent with this notion, the spectra obtained in the presence of PEG contained two components, one from the monomer and another from the phase-separated state. The monomer component could readily be subtracted using spectra obtained in the absence of PEG, and the corresponding spectra for the phase-separated state are shown in Fig. S1. When compared to the spectra of the monomers, the spectra for the phase-separated state have broader lines and lower amplitudes, indicative of reduced mobility (Fig. S1). Despite this reduction in mobility, the spectra of the phase-separated state were still relatively sharp. In fact, these spectra indicated a dynamic structure without pronounced and stable tertiary or quaternary contacts (44) and were characteristic for LLPS (45). Local mobility could be further quantified from the central line widths of the respective EPR spectra (Fig. S1), a measure that is inversely related to mobility. The line width for all spectra of the liquid condensate state was between 1.8 and 2.4 G. Such values are typical for disordered and highly dynamic regions in proteins. Interestingly, the largest line widths were observed for the sites in the polyQ (2.2 G and 2.4 G for 24R1 and 35R1, respectively), indicating a reduced mobility of the polyQ sites compared to those in the N17 or PRD. The polyQ region has previously been found to be a driver of liquid and solid phase separation for fluorescent protein-tagged Httex1 (36), and it is possible that the slightly larger line widths for residues in the polyQ reflect a larger proportion of the transient intermolecular contacts in the liquid condensate.

### Rapid liquid to solid transition of Httex1-RFP

**Figure 3:**
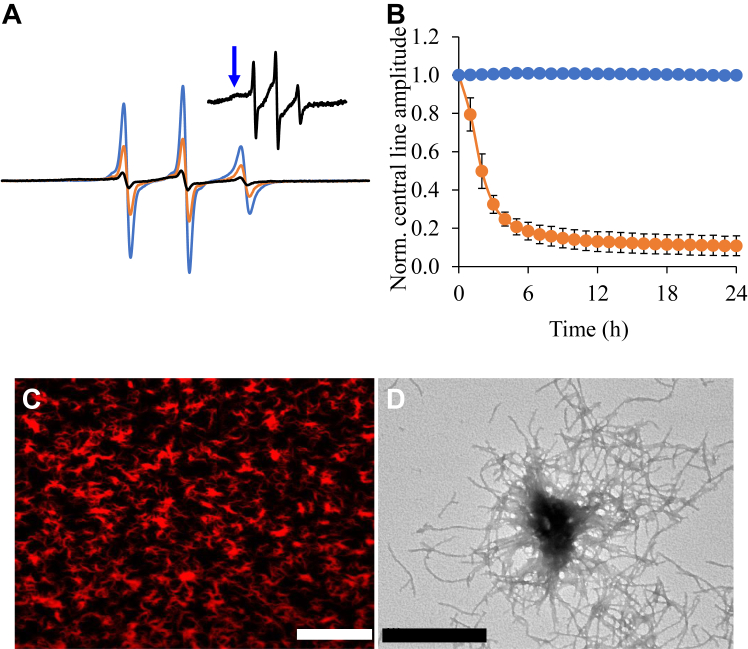
**Httex1(Q25)-RFP LLPS promotes formation of fibrils.***A*, EPR spectra showing the decrease of the EPR spectral amplitudes after adding 10% PEG to Httex1(Q25)-RFP-35R1. Spectra are shown for the 0 h (*blue*), 4 h (*orange*), and 24 h (*black*) time points after PEG addition. The *blue arrow* in the inset highlights the characteristic immobilized peak in the low field line of the EPR spectrum caused by fibril formation of Httex1(Q25)-RFP at 24 h. *B*, kinetic plot showing the decrease in the normalized central line amplitude of Httex1(Q25)-RFP-35R1 in the absence (*blue*) or presence of 10% PEG (*orange*). Error bars represent the standard deviation of the mean obtained from three independent measurements. The error bars for the *blue curve* are small and obscured by the size of the symbols. *C*, fluorescence microscopy image of Httex1(Q25)-RFP after 2 h of incubation with 10% PEG. The scale bar is 20 μm. *D*, electron microscopy image of Httex1(Q25)-RFP in the presence of 10% PEG obtained after 24 h. The scale bar is 1 μm. Protein concentration was 100 μM in all experiments. Images are representative of at least three independent experiments. Httex1, huntingtin exon-1; Httex1(Q25)-RFP, RFP-tagged Httex1 with 25Qs; LLPS, liquid–liquid phase separation; PEG, polyethylene glycol; RFP, red fluorescent protein.

Next, we tested whether LLPS impacts the formation of solid aggregates, such as fibrils. To resolve the kinetics of this process, we recorded EPR spectra of Httex1(Q25)-RFP-35R1 at different time points over a period of 24 h (Fig. 3, *A* and *B*). This derivative was chosen because sites in the polyQ are known to experience very pronounced immobilization and loss of amplitude upon fibril formation (24, 46), at a rate that matches that of ThT kinetics (21). As expected, a strongly immobilized spectral component emerged over time (Fig. 3*A*), and the concomitant drop in spectral amplitude was very rapid, becoming half-maximal in less than 2 h (Fig. 3*B*). These data were consistent with a rapid liquid-to-solid transition that is complete within a few hours. This transition also altered the appearance of the rounded, phase-separated structures, which became more irregular over time (Fig. 3*C*). Electron microscopy revealed that the solid aggregates were made up of fibrils formed by Httex1(Q25)-RFP (Fig. 3*D*). The liquid-to-solid transition was further investigated using fluorescence recovery after photobleaching (FRAP) experiments. First, we studied the large, rounded structures that formed immediately after sample preparation (Fig. S2). For these structures, recovery after photobleaching was fast, requiring only a few seconds, as expected for a liquid state (10). In contrast, recovery of more irregularly shaped aggregates, which formed over time, was barely detectable, as expected for solid aggregates. Thus, the FRAP data further supported the notion of a liquid-to-solid transition of Httex1(Q25)-RFP.

Next, we repeated the EPR kinetics for Httex1(Q25)-RFP-35R1 in the absence of PEG to test whether the lack of LLPS impacts the transition into fibrillar aggregates (Fig. 3*B*, *blue trace*). Under these conditions, the EPR amplitude did not change significantly, indicating that the transition into fibrils was very slow in the absence of LLPS (Fig. 3*B*). To further test the effect of LLPS on the fibril formation of Httex1(Q25)-RFP, we used 1,6-hexanediol, which has frequently been used to disrupt LLPS formation in a wide range of proteins (47), including Httex1-GFP (36). We found that 1,6-hexanediol also interfered with LLPS of Httex1(Q25)-RFP, as its addition largely disassembled round, phase-separated states, while increasing the red background fluorescence (Fig. S3, *A* and *B*). Importantly, this organic molecule strongly reduced the rate of fibril formation (Fig. S3*C*), further underscoring the importance of LLPS for the formation of solid Httex1(Q25)-RFP aggregates.

**Figure 4:**
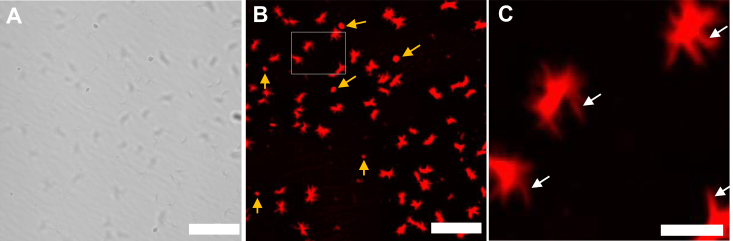
**Freshly prepared Httex1(Q39)-RFP rapidly transitions into fibrils.***A* and *B*, Httex1(Q39)-RFP immediately after mixing with 10% PEG was visualized by (*A*) differential interference contrast and (*B*) fluorescence microscopy. The *yellow arrows* highlight rounded phase separated structures. *C*, a zoomed in view of the *white* box from panel (*B*) shows irregularly shaped aggregates from which rod-like structures emanate (highlighted by *white arrows*). The protein concentration was 100 μM. The scale bars in (*A*) and (*B*) are 20 μm, and 5 μm in (*C*). Images are representative of at least three independent experiments. Httex1, huntingtin exon-1; Httex1(Q25)-RFP, RFP-tagged Httex1 with 25Qs; RFP, red fluorescent protein.

To test how longer Q-lengths affect phase separation, we generated Httex1(Q39)-RFP and imaged it in the absence or presence of PEG. Again, little LLPS could be observed in the absence of PEG, as irregularly shaped aggregates were visible sporadically, while most of the protein contributed to the diffuse red background (Fig. S4). In the presence of 10% PEG, however, some rounded, phase-separated structures were visible for Httex1(Q39)-RFP (Fig. 4, *A* and *B*, yellow arrows). These structures were reminiscent of those formed by Httex1(Q25)-RFP and suggested that RFP-tagged Httex1 with longer Q-lengths can also form liquid condensates. However, unlike what we observed for Httex1(Q25)-RFP, these structures were not the predominant species. When we quantified the number of such rounded structures, we found that they only amounted to ∼7% of the total phase-separated structures. Instead, most of the Httex1(Q39)-RFP aggregates (∼93%) formed more irregular structures, including rounded structures from which elongated, rod-like structures radiated outward (Fig. 4*C*, white arrows). Such structures have been observed before for Httex1(GFP) (36) and other proteins (10, 48) and are typically caused by the growth of solid aggregates (fibrils) from liquid condensates. Importantly, these structures were also similar to those observed for Httex1(Q25)-RFP after 2 h of incubation in the presence of PEG (Fig. 3*C*), a duration during which a little bit more than half of the fibril formation should have been completed (Fig. 3*B*, orange line). Together, these data suggested that RFP-tagged Httex1 (Httex1-RFP) with different Q-lengths exhibited qualitatively similar phase-separation behaviors, except that the liquid to solid transition was much faster for longer Q-lengths.

### Loss of RFP tag strongly reduces Httex1 propensity for phase separation

**Figure 5:**
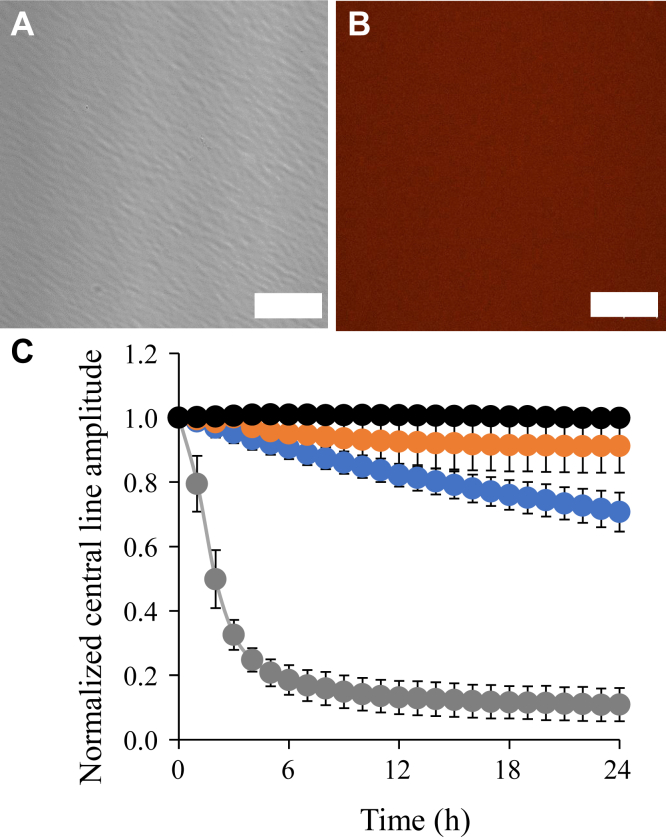
**Httex1(Q25) without RFP tag does not undergo LLPS.***A*, differential interference contrast image of untagged Httex1(Q25) at 100 μM in the presence of 10% PEG. *B*, fluorescence image of 97 μM unlabeled Httex1(Q25) and 3 μM Alexa-594 labeled Httex1(Q25) in the presence of 10% PEG. Scale bars are 20 μm. *C*, kinetic plots showing the decrease in the normalized central line amplitude of Httex1(Q25)-35R1 without PEG (*orange*), Httex1(Q25)-35R1 with PEG (*blue*), Httex1(Q25)-RFP-35R1 without PEG (*black*), and Httex1(Q25)-RFP-35R1 with PEG (*gra*y). Data for Httex1(Q25)-RFP-35R1 are reproduced from Figure 3*B* to facilitate comparison with the data obtained for the untagged protein. Error bars represent the standard deviation of the mean obtained from at least three independent measurements. The error bars for the *black trace* are smaller than the symbols. Images are representative of at least three independent experiments. Httex1, huntingtin exon-1; Httex1(Q25)-RFP, RFP-tagged Httex1 with 25Qs; LLPS, liquid–liquid phase separation; PEG, polyethylene glycol; RFP, red fluorescent protein.

To examine a potential role of the C-terminal RFP tag in phase separation, we performed parallel experiments on Httex1(Q25) without RFP tag. First, we used the DIC mode to visualize liquid condensate formation. Unlike Httex1(Q25)-RFP, the untagged protein did not give rise to any visible liquid condensates when viewed in DIC mode, even when crowding agent (10% PEG) was present (Fig. 5*A*). To enable detection *via* fluorescence, we repeated the experiments, but added 3% of Alexa-594-labeled Httex1(Q25) to the unlabeled protein. Again, little evidence of phase separation was found, as the red fluorescent signal was largely found in the diffuse background (Fig. 5*B*). In contrast, co-mixing of 3% Alexa-488-labeled Httex1(Q25)-RFP with Httex1(Q25)-RFP led to colocalization of the Alexa-488 and RFP signals, indicating that the chemical modification with an Alexa label did not prevent LLPS of the tagged protein (Fig. S5, *A* and *B*). Together, these results demonstrated that Httex1(Q25), without a C-terminal RFP tag, has no detectable propensity to form liquid condensates, even in the presence of PEG.

Next, we tested how the absence of the RFP tag affected fibril formation kinetics of Httex1(Q25). Kinetics were again obtained by monitoring the loss of EPR amplitude for a derivative that was labeled at position 35 in the polyQ region. As shown in Figure 5*C*, Httex1(Q25)-35R1 exhibited detectable aggregation in the presence as well as absence of PEG. A comparison of the aggregation kinetics of the tagged and untagged proteins revealed interesting differences. In the absence of PEG, where neither protein forms significant amounts of LLPS, the rate of fibril formation was much slower for Httex1(Q25)-RFP-35R1 (Fig. 5*C*, black trace, data for tagged protein are reproduced from Fig. 3*B* for better comparison) when compared to its untagged counterpart (Fig. 5*C*, orange trace). This indicated that the RFP tag was inhibitory in the absence of liquid condensate formation. The opposite result was observed in the presence of 10% PEG, wherein a significantly enhanced rate of aggregation was observed for Httex1(Q25)-RFP-35R1 relative to the untagged protein (Fig. 5*C*, gray and blue traces). These results further illustrated that LLPS of Httex1(Q25)-RFP potently promoted the formation of solid aggregates. In contrast to the tagged protein, the addition of 1,6 hexanediol only had a minor impact on the aggregation kinetics of Httex1(Q25) (Fig. S3*C*), further indicating that the aggregation of the untagged protein was not significantly mediated by LLPS.

### LLPS-mediated aggregation of Httex1(Q25)-RFP impacts aggregate structures *in vitro*

To test whether LLPS alters the structure of Httex1 aggregates, we used electron microscopy and EPR spectroscopy. It has previously been reported that Httex1 aggregation in the absence of LLPS proceeds *via* small oligomeric as well as short and skinny fibrils (21), which then grow longer and thicker over time (41). In agreement with these studies, fibrils of the untagged Httex1(Q25) were relatively small after 1 h, gradually increasing in size after 24 h (Fig. S6, *A* and *B*). In stark contrast, the LLPS-mediated aggregation of Httex1(Q25)-RFP resulted in fibrils that were already much wider and more bundled after only 1 h (Fig. S6*C*) than those of the untagged protein after 24 h. Together with the fluorescence microscopy image (Fig. 3*C*), these results suggested that entire fibril bundles grow out of the condensates.

Next, EPR was used to investigate local structural differences in the respective fibrils. Toward this end, we generated RFP-tagged and untagged Httex1(Q25) fibrils that were labeled in the polyQ (35R1) or the N17 (7R1). The spectra of the 35R1 derivatives were nearly identical for the Httex1(Q25) and Httex1(Q25)-RFP fibrils (Fig. S7*A*). In both cases, the spectra were dominated by strong immobilization, suggesting that the polyQ forms the fibril core in Httex1(Q25) and Httex1(Q25)-RFP fibrils. In contrast, EPR spectral differences were observed for 7R1, which was largely immobilized in Httex1(Q25) fibrils, but which exhibited a more mobile component in Httex1(Q25)-RFP fibrils (see arrow in Fig. S7*B*). Overall, these data indicated that LLPS-mediated aggregation of the RFP-tagged protein altered fibril morphology and affected the local structure in the N17 region.

### RFP tag alters aggregation in cells

**Figure 6:**
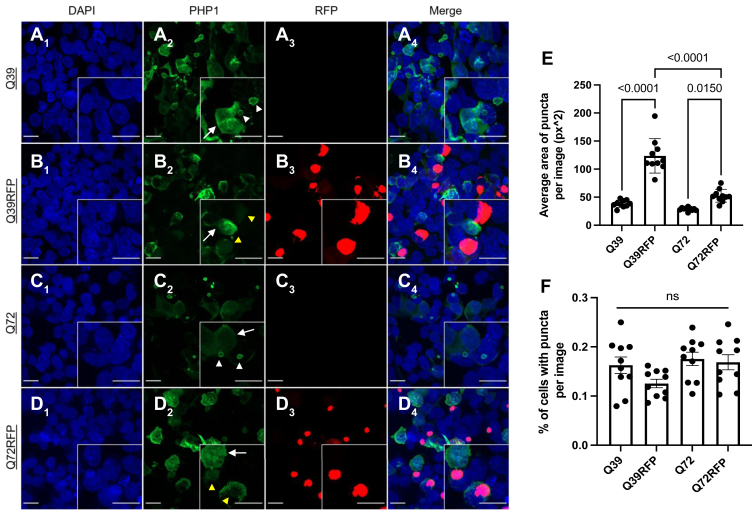
**RFP tag alters the puncta size and pattern of mutant Httex1 antibody labeling.***A*–*D*, representative confocal images of untagged Httex1(Q39) (*A*), Httex1(Q39)-RFP (*B*), untagged Httex1(Q72) (*C*), and Httex1(Q72)-RFP (*D*) transfected HEK293T cells. Cells expressing Httex1(Q39) or Httex1(Q72) with or without RFP were fixed 48 and 24 h after transfection, respectively, and show distinct puncta formation (as seen by the RFP channel and differential interference contrast images [not shown]). The nucleus was counterstained with DAPI, and Httex1 aggregates were detected using a specific antibody against mutant huntingtin, PHP1, and visualized with Alexa Fluor 488 conjugated secondary antibody. Insets on the lower righthand corner of each image show puncta formation within cells at higher magnification. *White arrowheads* indicate PHP1 reactivity toward untagged Httex1(Q39) and Httex1(Q72) puncta, *yellow arrowhead* indicate PHP1 reactivity toward RFP-tagged puncta, and *white arrows* indicate cells without aggregates. Scale bars are 20 μm. *E*, image-based quantification of the average area of the puncta per image (n = 10) and *F*, percent of cells with puncta in each image (n = 10) across groups was done using FIJI, and PRISM was used to generate graphs and perform statistical analysis (One-way ANOVA, Tukey’s multiple comparison test). Httex1, huntingtin exon-1; RFP, red fluorescent protein.

Having established the aggregation-altering properties of the carboxyl-terminal RFP tag on Httex1 *in vitro*, we next tested its effect in cells. First, we performed live-cell imaging of HEK293T cells transfected with Httex1(Q72)-RFP to determine the time course of aggregation (Fig. S8). Httex1(Q72)-RFP fluorescence was first imaged 11 h post-transfection. Initially, the fluorescence was diffusely cytoplasmic. Then, within a 15 min window between frames, the fluorescence rapidly coalesced into a round punctum. This time course was remarkably rapid, especially when compared to the kinetics of fibril formation in Figures 3 and 5, potentially indicating an involvement of LLPS in the initial punctum formation. Notably, transmission electron microscopy showed that puncta formed by a similar construct, Httex1Q72-GFP, are membrane-less (49), supporting the notion that these puncta are not macroautophagy structures which are enveloped by double membranes (50) Next, HEK293T cells expressing untagged or RFP-tagged Httex1 with two different pathogenic polyQ lengths, Httex1(Q39) and Httex1(Q72), were fixed after sufficient time passed for the cells to form puncta. In the case of Httex1(Q39), cells showed puncta formation by 48 h, whereas Httex1(Q72) showed distinct puncta formation by 24 h, which is consistent with the notion that longer Q-lengths have faster aggregation kinetics (51). In order to examine the effect of the RFP tag on mutant Httex1 expression, untagged Httex1(Q39), Httex1(Q39)-RFP, untagged Httex1(Q72), and Httex1(Q72)-RFP transfected cells were labeled with PHP1 antibody, which has strong reactivity to fibrillar Httex1 and monomeric forms to a lesser degree (52). As shown in Figure 6, diffuse cytoplasmic staining reflecting reactivity to monomeric or smaller oligomeric Httex1 can be seen in some cells without aggregates (Fig. 6 A_2_-D_2_, white arrows). This diffuse PHP1 signal overlaps with that of diffuse Httex1(Q39)-RFP and Httex1(Q72)-RFP signal, which requires a higher signal threshold for visualization due to their weak signal intensity relative to that of formed puncta (Fig. S9). Thus, PHP1 binds strongly to monomeric and/or small oligomeric forms of Httex1-RFP, but less to its aggregates. With regard to aggregated Httex1, the pattern of PHP1 labeling appeared to be qualitatively different in Httex1(Q39) and Httex1(Q72) compared to their RFP-tagged analogs. More specifically, in the untagged variants, PHP1 showed strong immunoreactivity to the outer shell or periphery of the puncta (Fig. 6 A_2_ and C_2_, white arrowheads), whereas in Httex1(Q39)-RFP and Httex1(Q72)-RFP, PHP1 showed diffuse and weak reactivity to puncta (Fig. 6 B_2_ and D_2_, yellow arrowheads). This pattern of reactivity indicates that the RFP-tagged puncta have a different microenvironment than untagged puncta. In addition to the distinct pattern of antibody reactivity, another key difference that the addition of an RFP tag conferred to cells expressing Httex1(Q39) and Httex1(Q72) was puncta size. Using overlays of the RFP channel, PHP1 channel, and DIC channel, the average area of puncta across images was quantified. This image-based quantification revealed significant differences in puncta size across groups, with the Httex1(Q39)-RFP and Httex1(Q72)-RFP showing significantly larger puncta area on average than Httex1(Q39) or Httex1(Q72) (Fig. 6*E*). Httex1(Q39)-RFP also showed significantly larger puncta than Httex1(Q72)-RFP. While the puncta size was affected by the addition of an RFP tag, the number of puncta was not affected, as evidenced by the fact that the percentage of cells with puncta per image remained nonsignificant across groups (Fig. 6*F*). In contrast to the proteins with pathogenic Q-lengths, expression of Httex1(Q16)-RFP gave rise to largely diffuse background fluorescence (Fig. S10), consistent with prior studies, which suggested that longer Q-lengths promote LLPS as well as fibril formation (36, 39).

## Discussion

Huntingtin aggregation and toxicity have been widely studied using fluorescent protein tags (35, 36, 40, 53, 54, 55, 56, 57, 58). These tags allow for a convenient way to monitor liquid and solid phase transitions of Httex1 in real time. In fact, much of what we know about Httex1 aggregation, including its ability to undergo LLPS (36, 37, 39), has come from such fusion proteins. However, the effect of these tags on phase separation and aggregation of Httex1 had not been investigated. Here, we examined the influence of a widely used fluorescent protein tag, RFP, on the LLPS and aggregation properties of Httex1 for both pathogenic and nonpathogenic polyQ lengths. The RFP tag strongly promoted LLPS, which, in turn, accelerated and altered fibril formation. Overall, LLPS of the RFP-tagged protein was greatly enhanced by crowding agents which mimic the crowded cellular environment. No obvious LLPS was observed for the untagged Httex1(Q25) protein, even when a crowding agent was added. Thus, a fluorescent protein tag, either an RFP tag or a GFP tag as used in prior studies (36, 37), strongly promotes LLPS *in vitro*. Considering that many studies in the past have used overexpression of GFP- or RFP-tagged proteins, it is possible that exaggerated amounts of LLPS might have been introduced in prior studies. Puncta formation is commonly used to look for aggregates. However, based on the present data and those of others (36, 37), it is quite possible that at least some of these puncta formed by the tagged Httex1 derivatives might have been more liquid-like.

**Figure 7:**
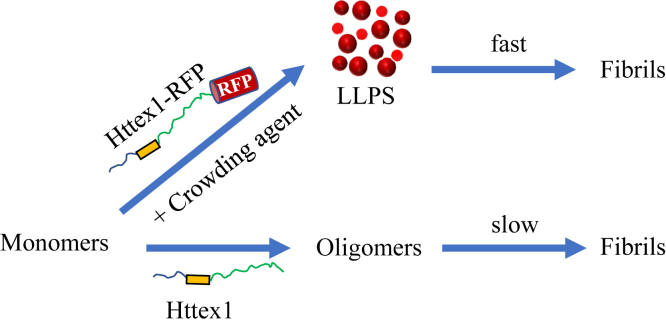
**Schematic representation of the influence of RFP on Httex1 phase separation and aggregation.** Two aggregation pathways are identified *in vitro*. In one pathway, fibril formation is mediated by LLPS. This pathway is only accessible to Httex1-RFP in the presence of crowding agents, and it causes very rapid fibril formation. The other pathway is the previously identified misfolding process in solution which proceeds *via* smaller oligomeric species. This is the main pathway for untagged Httex1, while it was very slow for Httex1-RFP (Fig. 3*B*, blue trace). The *blue line* in the Httex1 represents the N17 region, the *orange box* represents the polyQ, and the *green line* represents the PRD. Httex1-RFP, RFP-tagged Httex1; LLPS, liquid–liquid phase separation; PRD, proline-rich domain; RFP, red fluorescent protein.

Tag-induced LLPS also enhanced the formation of solid-like aggregates, such as fibrils. In the case of Httex1(Q25)-RFP, aggregation into fibrils was undetectable in the absence of PEG during the duration of the experiment, whereas LLPS using PEG resulted in very rapid fibril formation. This fibril formation was much faster than that of the untagged protein, which did not yield any detectable LLPS and only saw a modest increase in aggregation kinetics upon addition of PEG. Aggregation was even faster for Httex1(Q39)-RFP, where fibrils were found to emanate from LLPS already in freshly prepared sample. This is consistent with recent yeast studies using GFP-tagged Httex1, which suggested that longer Q-lengths not only promote LLPS but that they also rapidly transition into solid-like aggregates, making it increasingly harder to detect the more short-lived liquid-like aggregates for proteins with expanded Q-lengths (36). Together, these data suggest that fluorescent protein tagging of Httex1 might artificially promote LLPS in cells, especially at high concentrations present under conditions of overexpression (Fig. 6). Moreover, LLPS altered the aggregation pathway and accelerated the formation of solid aggregates (Fig. 7). The fibrils formed under LLPS conditions from recombinant proteins were also much more bundled, with altered N-terminal structure. In support of a potential effect of the RFP tag under physiological conditions, we also found that tagging had a significant impact on aggregate size and antibody staining in cells. Interestingly, larger aggregates were again observed for the tagged proteins. In addition, it has recently been reported that fluorescently tagged Httex1 has a different interactome in the cell (49). Therefore, it is possible that fluorescent tags and LLPS might also affect toxicity.

While our data indicate that caution is needed in interpreting the results from overexpression of fluorescent protein-tagged Httex1, we cannot categorically exclude that untagged Httex1 or other N-terminal huntingtin fragments might undergo LLPS under physiological conditions. First, it is possible that proteins or cofactors might promote the transition of huntingtin fragments into liquid condensates. Second, the LLPS promoting effects of the bulky, C-terminal RFP could be mimicked by natural huntingtin sequences that are found C terminally of Httex1 in some of the longer huntingtin fragments. Longer N-terminal fragments of huntingtin have indeed been found in the inclusions of diseased brain (59, 60, 61), and they could be of pathogenic relevance. Moreover, it has been shown that longer N-terminal fragments form aggregates that are different from those of Httex1, and a recent study also demonstrated that Htt(1–586) can undergo LLPS in the presence of dextran at 20 μM concentrations (38). Third, it is possible that longer Q-lengths could allow untagged Httex1 to take up transient LLPS, even though a rapid liquid-to-solid transition might make such structures more difficult to resolve. Future work will be needed to resolve the extent to which such factors can promote LLPS in untagged Httex1. Moreover, it will be important to understand the mechanisms by which fluorescent protein tags or additional protein sequences promote LLPS.

EPR data also provided some structural insights into the liquid-like state. When compared to the corresponding spectra of the monomer, the spectra of the LLPS state were slightly broadened, indicating that LLPS reduces the mobility relative to the free monomer. However, the spectra were still relatively sharp showing that mobility remained high in the liquid-like phase separated state. In fact, these spectra were consistent with a largely disordered structure devoid of stable tertiary or quaternary protein contacts. While the spectra from sites in different domain were qualitatively similar, subtle differences were found. The broadest lines were obtained for sites in the polyQ regions, perhaps indicating that the polyQ engages in more transient interactions that stabilize LLPS. This notion would be consistent with the finding that longer Q-lengths promote LLPS in yeast (36).

One of the central findings of the present study is that the addition of a fluorescent protein tag can significantly alter its LLPS and aggregation behavior for Httex1. Such tags are commonly used for monitoring phase separations of numerous proteins, as fluorescence provides a convenient way to detect phase separations in real time. At a minimum, the potential effects of such protein tags need to be examined to ensure that they do not alter the phase separation or aggregation propensity of a given protein of interest.

## Experimental procedures

### Protein expression and purification

Thioredoxin-tagged Httex1 with or without RFP at the C terminus was expressed in *E. coli* BL21 (DE3) cells as described previously (21, 62). We used mRFP1 that is designed to exist in monomeric form (63). Briefly, *E. coli* BL21 cells transformed with Httex1 in pET28b were grown overnight at 37 ^°^C, followed by 50-fold dilution into LB media. Protein expression was induced using 1 mM IPTG followed by incubation at 16 ^°^C for 48 h. The bacterial pellets were harvested by centrifugation at 3500*g* for 30 min. Bacteria were lysed in 20 mM Tris pH 7.4 containing 20 mM imidazole, 300 mM NaCl, and 1% Triton-X using a tip sonicator. The lysate was centrifuged at 18,000*g* for 30 min, and the supernatant was incubated with NiHis60 (Takara Bio) for 30 min. The protein was eluted using 20 mM Tris pH 7.4 with 300 mM NaCl and 300 mM imidazole. For EPR and fluorescence experiments, cys mutants were used for covalent linkage of the respective reporter groups. In case of cys mutants, 1 mM DTT was added to the lysis buffer to prevent any disulfide bond formation. The DTT was washed away using washing buffer (20 mM Tris pH7.4, 300 mM NaCl, 50 mM Imidazole) prior to the elution of the protein from the NiHis60 beads. The cys mutants were then labeled with 5-fold molar excess of MTSL (Toronto Research Chemicals) or Alexa Fluor 488, 594 (Thermo Fisher Scientific). The excess MTSL or Alexa Fluorophore was removed using a HiTrap QXL anion exchange column (Cytiva Life Sciences). Httex1(Q25) was labeled with Alexa-594, and Httex1(Q25)-RFP was labeled with Alexa-488 at position 80 in the PRD. The spin labeling positions for EPR are given in the text and legends. Thioredoxin-tagged Httex1 was then treated with enterokinase (EKmax) (Thermo Fisher Scientific) for 30 min at 32 ^°^C to remove thioredoxin. Subsequent purification was performed using a C4 reversed phase column for Httex1 without RFP, while the Httex1-RFP was purified using a HiTrap QXL anion exchange column.

### Fluorescence microscopy

Concentrated stocks of Httex1 with or without RFP were diluted into the required concentrations in 20 mM TBS with 10% PEG 4000. Samples were then imaged using a Zeiss LSM 800 upright confocal microscope at 63x. For the untagged Httex1 without RFP, we mixed 3 μM Alexa-594-labeled Httex1 with 97 μM unlabeled Httex1. The quantification of Httex1(Q39)-RFP phase separation images was performed using Fiji Image J software by analysis of 450 structures. Rounded structures were identified using a circularity value ≥0.9, while shapes with values below 0.9 were considered irregular. In case of comixing experiments where Httex1(Q25)-RFP was labeled with Alexa-488, the green channel filter parameters were adjusted to avoid any bleed through contribution of Httex1 (Q25)-RFP alone.

### FRAP measurements

FRAP of Httex1(Q25)-RFP LLPS was carried out using Leica SP8 DIVE multiphoton confocal fluorescence imaging system at 63x oil-immersion objective powered by a Chameleon Discovery laser at 1050 nm (Coherent) and DMi8 inverted microscope’s external Leica 4Tune spectral hybrid detectors (emission at 580 nm for RFP fluorescence). A series of 10 prebleach images was captured, followed by photobleaching of a region of interest for ∼2.5 s at 100% laser power, and then capturing images every ∼260 milliseconds for 20 s after photobleaching. The analysis of FRAP data was performed in Fiji using Stowers ImageJ plugins (https://research.stowers.org/imagejplugins/zipped_plugins.html). The intensity obtained from Fiji was normalized to the prebleach image intensity, which was set to 1.

### EPR spectroscopy

Samples were loaded in borosilicate capillaries with a 0.6 mm inner diameter. The CW-EPR spectra of freshly prepared spin-labeled Httex1(Q25)-RFP in the absence or presence of 10% PEG were recorded at 200 μM on an X-band Bruker EMX EPR spectrometer fitted with a Bruker ER4119HS resonator at room temperature. An incident power of 12.7 mW along with a scan width of 100 G was used. For kinetic measurements, the central line amplitudes were obtained using WinAcquisit software. The protein concentration for kinetic measurements was 100 μM. Difference spectra were obtained using the EPR 130 program, and the same program was also used for normalization *via* double integration. All measurements were performed at room temperature.

### 1,6-Hexanediol dissolution of liquid condensates

Condensates of Httex1 (Q25)-RFP in the presence of 10% PEG were prepared as above and imaged using time lapse movies with a frame rate of 1 s on a Zeiss LSM 800 upright confocal microscope with 63x oil objective. 1,6-hexanediol in 20 mM Tris pH 7.4, 150 mM NaCl, 10% PEG was then added to yield a final concentration of 10% 1,6-hexanediol during the image acquisition to record the dissolution of the LLPS over time.

### Transmission electron microscopy

The samples were diluted 10-fold and deposited on carbon coated copper EM grids and stained with 1% uranyl acetate. The negative stain images were acquired on a JEOL-JEM 1400 transmission electron microscope. Httex1 (Q25) and Httex1 (Q25)-RFP fibril widths were quantified using Fiji ImageJ.

### Cell-based assay

HEK293T cells (Obtained from ATCC) were split and seeded onto 24-well plates with poly-D-lysine-coated coverslips and grown to 70% confluency. Cells were transfected with 500 ng of Httex1(Q39), Httex1(Q39)-RFP, Httex1(Q72), or Httex1(Q72)-RFP plasmid DNA (Genscript) using Lipofectamine LTX with Plus Reagent transfection kit (Thermo Fisher Scientific), according to manufacturer’s protocol. Cells were allowed to express constructs for 24 h (Httex1(Q72) and Httex1(Q72)-RFP) or 48 h (Httex1(Q39) and Httex1(Q39)-RFP), after which the cells were fixed with 3.7% formaldehyde, permeabilized with 0.1% Triton-X, and blocked with 1% bovine serum albumin for 1 h at room temperature. To label the Httex1 aggregates, cells were incubated with mouse PHP1 primary antibody (EMD Millipore) at 1:500 in 0.1% bovine serum albumin blocking buffer overnight at 4 °C, followed by incubation with Alexa Fluor 488-conjugated donkey anti-mouse antibody (Invitrogen) at 1:200 in same block for 1 h at room temperature. Coverslips with cells were mounted onto glass micro slides with Vectashield antifade mounting medium with DAPI (Vector Laboratories) and imaged with Zeiss LSM 800 confocal microscope. Image analysis was performed using FIJI and Prism software. Fiji was used to manually count the number of cells, number of puncta, and area of puncta in each image (total 10 images per group). Prism was used to generate graphs and do statistical analysis.

## Data availability

All data are contained within the manuscript.

## Supporting information

This article contains supporting information.

## Conflict of interest

The authors declare that they have no conflicts of interest with the contents of this article.
