# Supplementary material for: Fluorescent protein tagging promotes phase separation and alters the aggregation pathway of huntingtin exon-1
Source: J Biol Chem. 2023 Dec 21;300(1):105585. doi: 10.1016/j.jbc.2023.105585 (PMC10825056; doi:10.1016/j.jbc.2023.105585)
Supplement: Supporting Figures S1–S10 [file mmc1.docx]

**Supporting information**

**
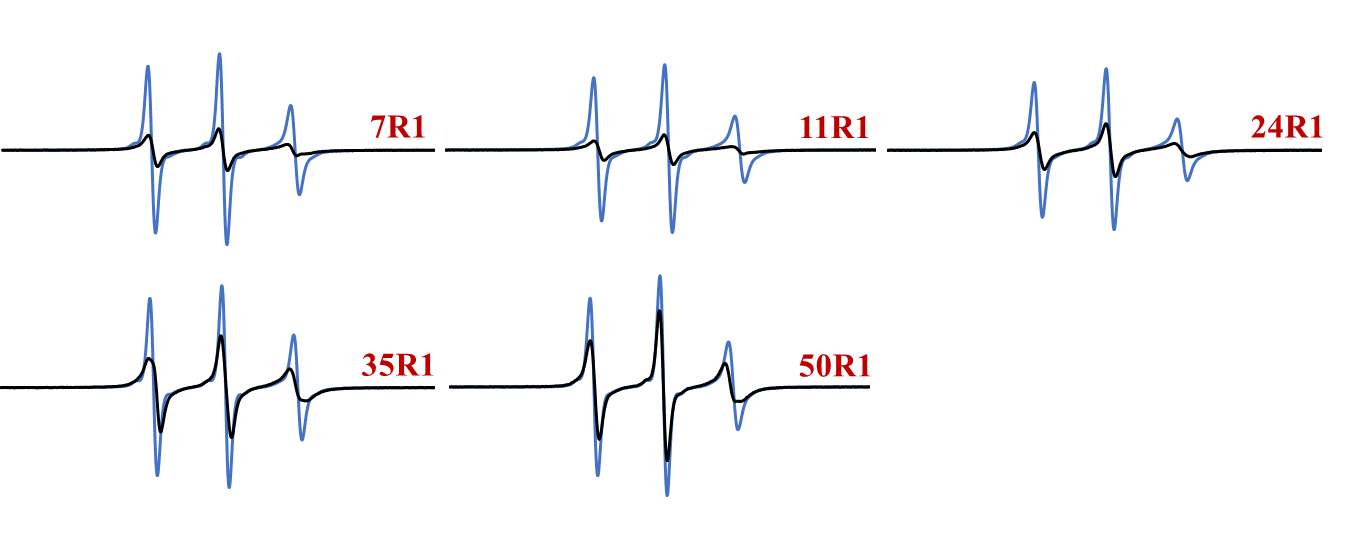
**

**Supporting Fig. S1. Liquid-like phase separated state of Httex1(Q25)-RFP has reduced dynamics when compared to the free monomer.** The X-band EPR spectra of the LLPS state of Httex1(Q25)-RFP (obtained from difference spectra of Httex1(Q25)-RFP in the absence and presence of 10% PEG, Fig. 2) are shown in black, while the spectra of monomeric Httex1(Q25)-RFP (in the absence of PEG) are in blue. All spectra were normalized to the same number of spins. Spectra are representative of at least three independent measurements.


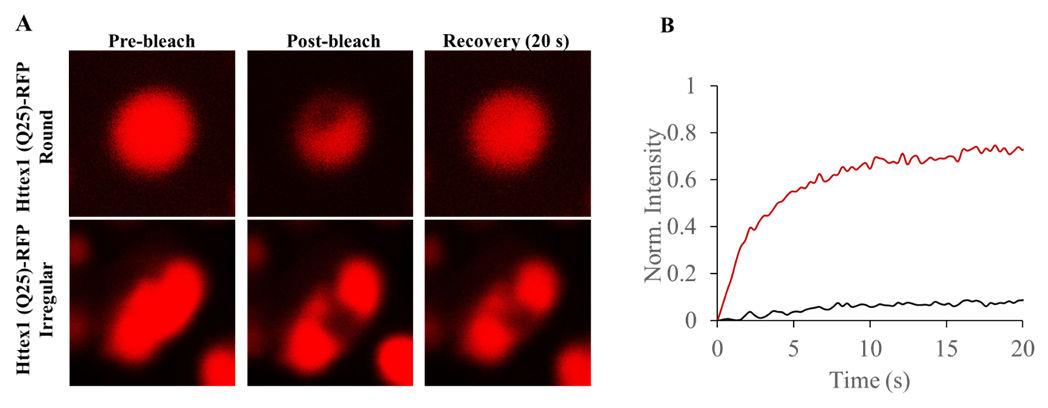


**Supporting Fig. S2. FRAP (Fluorescence recovery after photobleaching) of round and irregular shaped phase separated Httex1(Q25)-RFP.** (A) Representative images obtained during FRAP experiments of round and irregular shaped assemblies showing pre-bleach, post-bleach and recovery (after 20 s) images, (B) FRAP recovery curves for round (red) and irregularly shaped (black) assemblies shown in panel A. Scale bars are 1 µm.

**
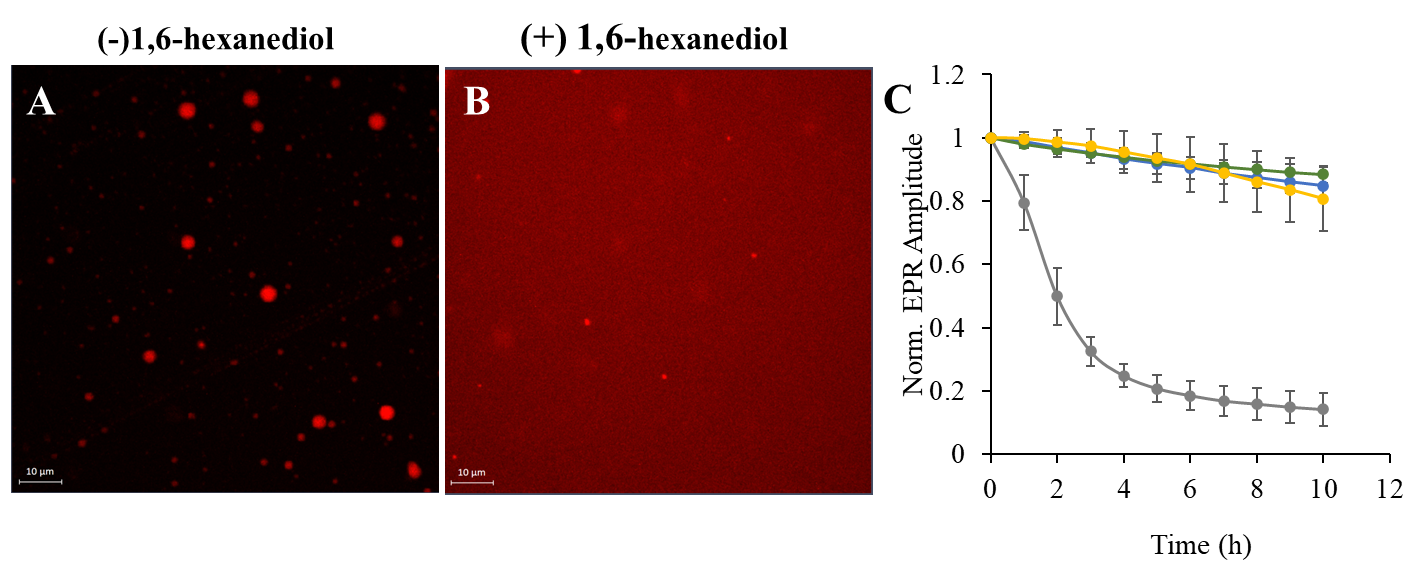
**

**Supporting Fig. S3. 1,6-hexanediol inhibits LLPS of Httex1(Q25)-RFP and strongly reduces LLPS mediated fibril formation.** Fluorescence microscopy images of freshly prepared Httex1(Q25)-RFP in the presence of 10% PEG (A) before and (B) after addition of 1,6-hexanediol. (C) EPR kinetics of Httex1(Q25)-35R1 and Httex1(Q25)-RFP-35R1 aggregation in the absence and presence of hexanediol. The green trace is for Httex1 (Q25)-RFP-35R1 with 5% hexanediol and 10% PEG, while the corresponding curve obtained in the absence of 1,6-hexanediol is in gray. The yellow trace shows the results from Httex1(Q25)-35R1 with 5% hexanediol and 10% PEG and the results obtained in the absence of 1,6-hexanediol are shown in blue. The scale bar is 10 µm in A and B. Images shown are representative of at least three independent experiments. Error bars represent the standard deviation of the mean obtained from at least three independent measurements.

**
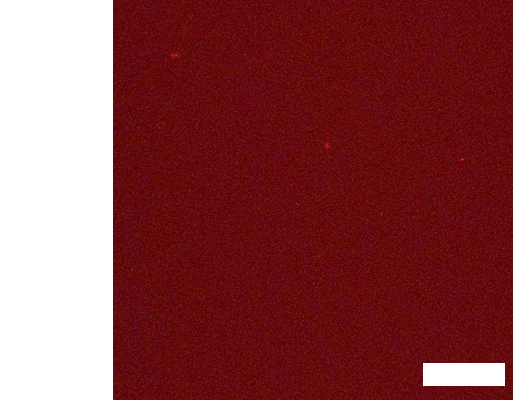
**

**Supporting Fig. S4. Httex1(Q39)-RFP shows little evidence of LLPS in the absence of crowding agents.** Freshly dissolved Httex1(Q39)-RFP was imaged using fluorescence microscopy. Scale bar is 20 µm. Images are representative of at least three independent experiments.

**
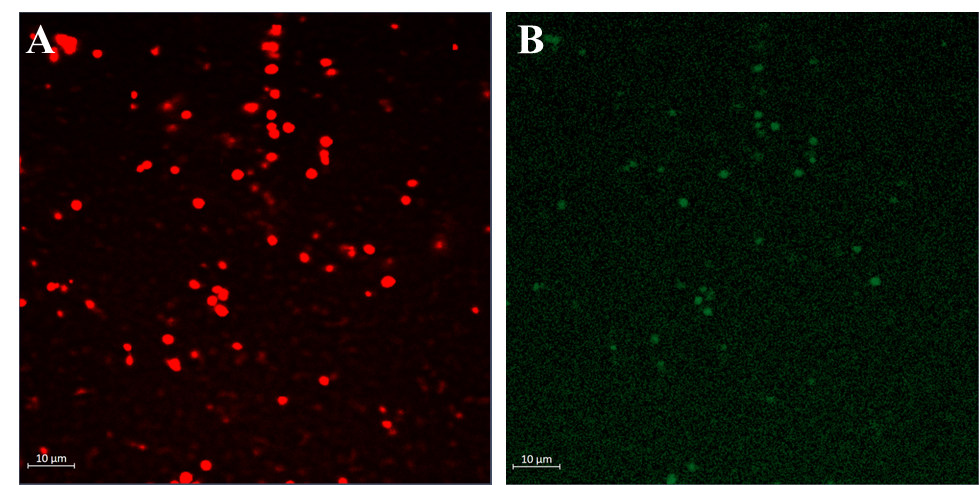
**

**Supporting Fig. S5. Alexa-488 labeling does not affect Httex1(Q25)-RFP LLPS.** Fluorescence microscopy images of 97 µM Httex1(Q25)-RFP and 3 µM Alexa-488 labeled Httex1(Q25)-RFP in the presence of 10% PEG were visualized using (A) the RFP signal in the red channel and (B) the Alexa-488 signal in the green channel. Scale bar is 10 µm. Images are representative of at least three independent experiments.

**
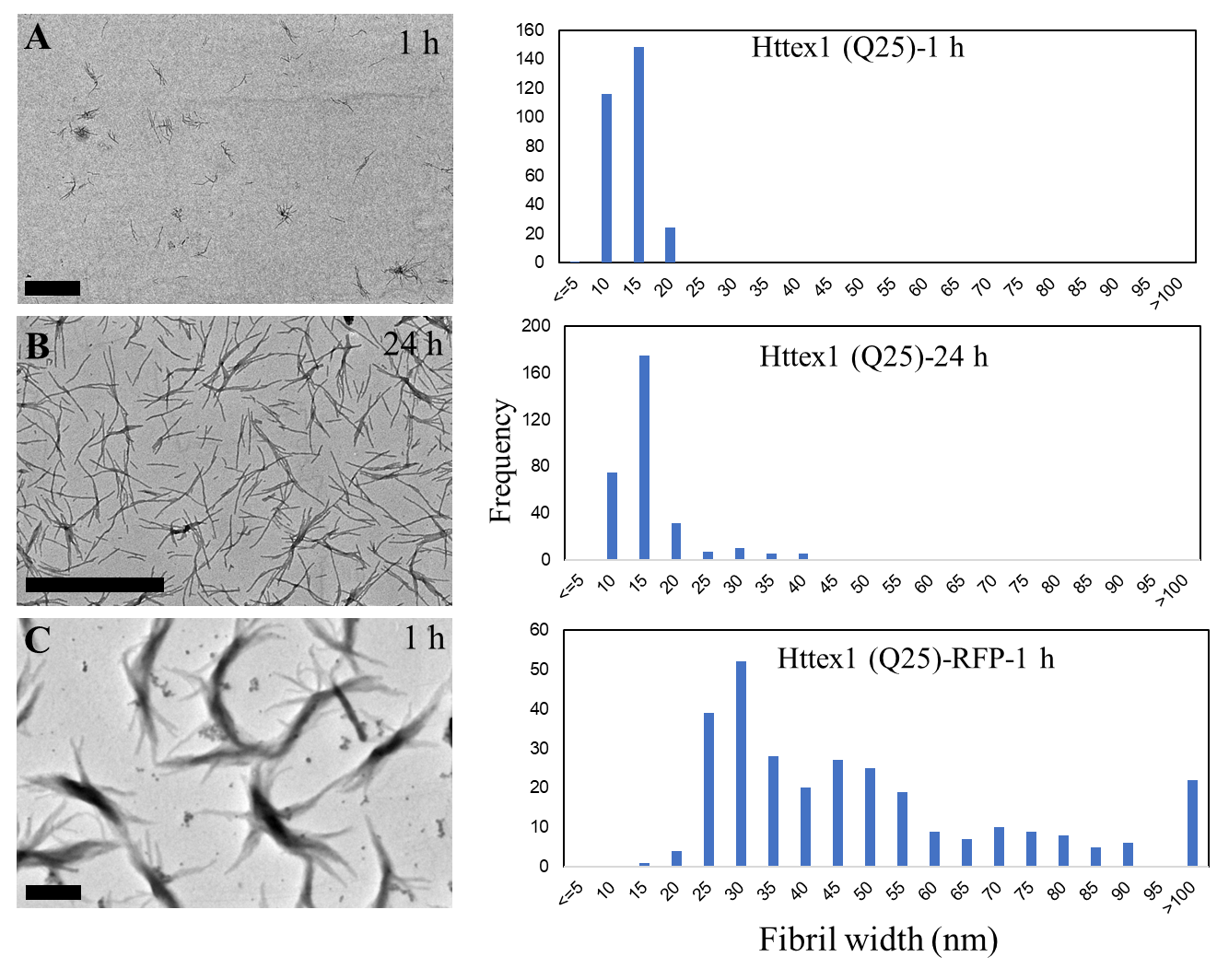
**

**Supporting Fig. S6. Comparison of fibril morphology of Httex1(Q25) and Httex1(Q25)-RFP.** Transmission electron microscopy images of fibrils from Httex1(Q25) incubated in 10% PEG obtained after (A) 1 hour or (B) 24 hours of incubation. (C) The width of Httex1(Q25)-RFP fibrils after 1 hour is already much larger than that of the untagged protein after 24 hours (B). The respective width distributions are shown in the adjacent right panel. Scale bar is 1 µm. Images are representative of at least three independent experiments.

**
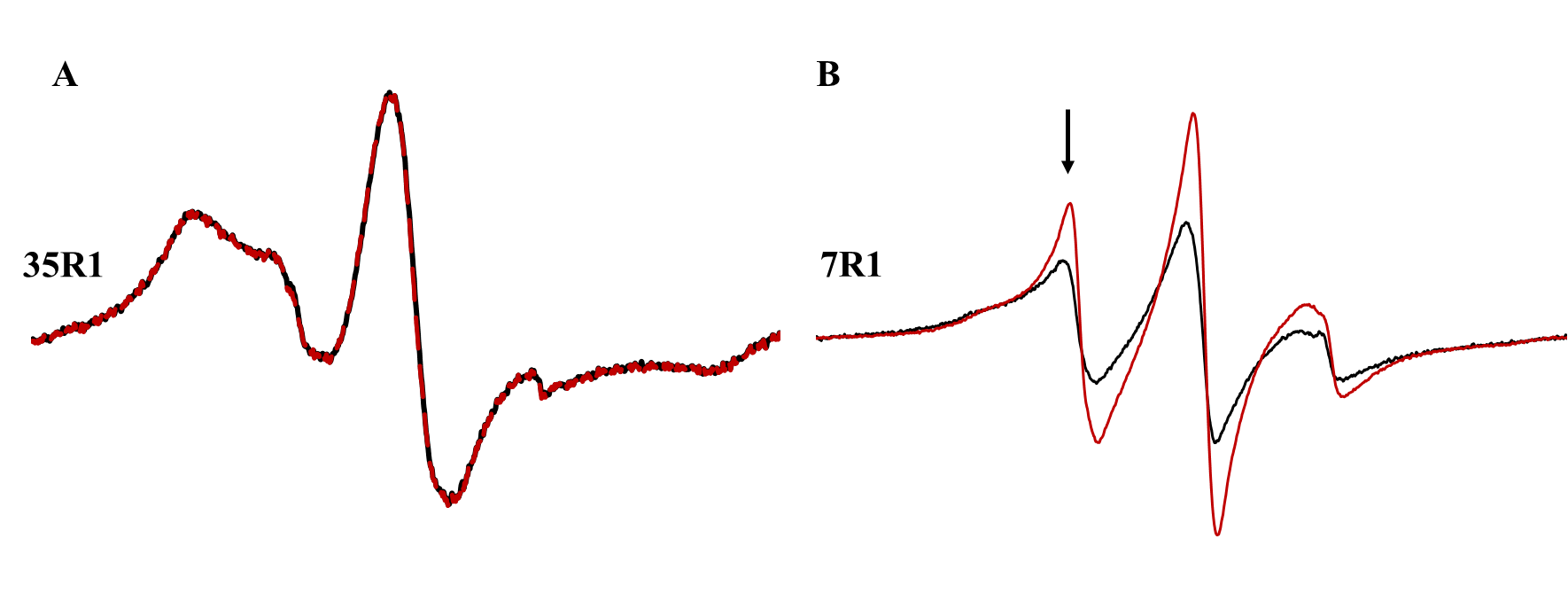
**

**Supporting Fig. S7. EPR spectra of Httex1(Q25) and Httex1(Q25)-RFP fibrils reveal structural differences in the N17.** Continuous wave X-band EPR spectra of Httex1(Q25) (black) and Httex1(Q25)-RFP (red) fibrils, spin-labeled at (A) at residue 35 (35R1, polyQ site) or (B) at residue 7 (7R1, N17 site). Spectra are representative of at least three independent measurements. EPR spectra for a given labeling positions are normalized to the same number of spins.

**
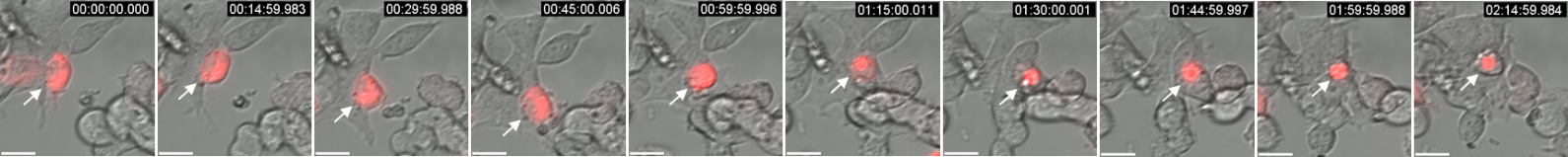
**

**Supporting Fig. S8. Live-cell imaging showing diffuse cytoplasmic Httex1-RFP fluorescence coalescence into a bright punctum within a cell.** HEK293T cells expressing Httex1(Q72)-RFP were imaged using time-lapse microscopy starting 11 h after transfection. A representative panel of time-lapse images shows Httex1(Q72)-RFP signal in one cell (white arrow) from t=0 min when imaging was initiated and acquired every 15 min thereafter. Live-cell imaging was acquired with Leica Thunder widefield microscope and time-lapse images were created using LAS X software. Scale bars are 20 µm.


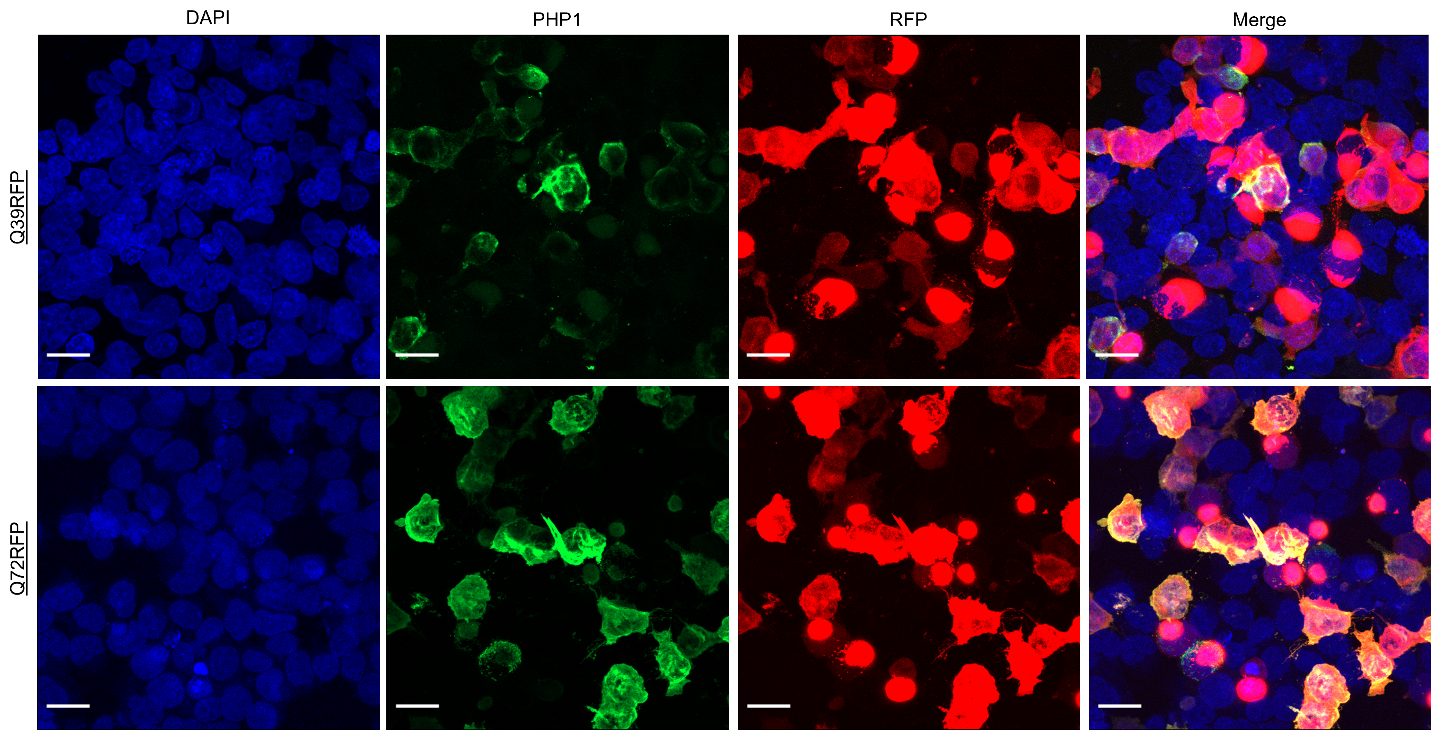


**B_4_**

**B_3_**

**B_2_**

**B_1_**

**A_3_**

**A_4_**

**A_2_**

**A_1_**

**Supporting Fig. S9.** Representative panel showing the same images used in Figure 6 for RFP-tagged Q39 and RFP-tagged Q72 with increased RFP signal to show both the aggregated and diffuse Httex1 labeling in cells. (A) Httex1(Q39)-RFP, and (B) Httex1(Q72)-RFP transfected HEK293T cells. Scale bars are 20 µm.

**
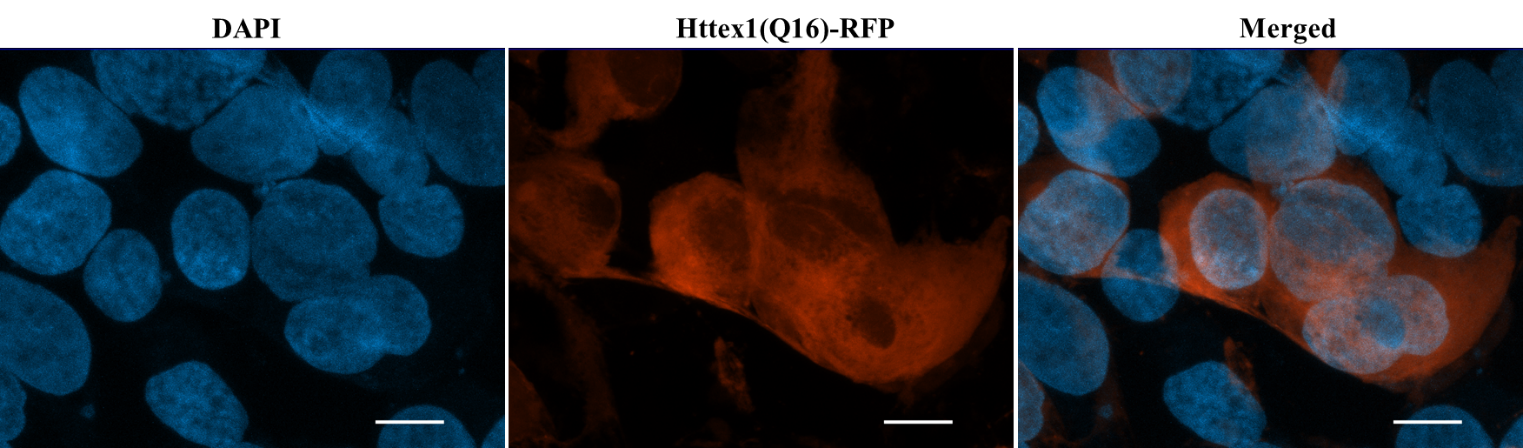
**

**Supporting Fig. S10. Diffuse RFP signal seen in cells expressing short non-pathogenic Q-length.** HEK293T cells expressing Httex1(Q16)-RFP were fixed 24 h post-transfection, counterstained with DAPI and imaged using Zeiss LSM 800 Confocal Laser Scanning Microscope. Scale bars are 10 µm.
